# Barriers to addiction treatment among formerly incarcerated adults with substance use disorders

**DOI:** 10.1186/s13722-018-0120-6

**Published:** 2018-08-21

**Authors:** Mandy D. Owens, Jessica A. Chen, Tracy L. Simpson, Christine Timko, Emily C. Williams

**Affiliations:** 1 Health Services Research and Development, VA Puget Sound Health Care System, Department of Health Services, University of Washington, 1660 South Columbian Way, S-152, Seattle, WA 98108 USA; 2 Center for Substance Abuse Treatment and Education, VA Puget Sound Health Care System, Seattle, USA; 3 Department of Psychiatry and Behavioral Sciences, University of Washington, Seattle, WA USA; 4 Center for Innovation to Implementation, VA Palo Alto Health Care System, Palo Alto, USA; 5 Department of Psychiatry and Behavioral Sciences, Stanford University, Palo Alto, CA USA

**Keywords:** Barriers, Treatment, Substance use disorder, Jail, Inmates, Prisoners, Alcohol, Drugs

## Abstract

**Background:**

Addiction treatment improves substance use and criminal recidivism outcomes among justice-involved individuals with substance use disorders, but is underutilized. Although information exists regarding barriers to addiction treatment among individuals with substance use disorders more generally, less is known about barriers among individuals with previous justice involvement. The purpose of this pilot study was to describe barriers to addiction treatment in a sample of adults with a substance use disorder who participated in a pilot trial of brief interventions and were recently released from jail.

**Methods:**

Incarcerated individuals who were arrested for an alcohol- or drug-related crime and reported moderate or high alcohol use on the ASSIST (n = 28; 96.4% men) were recruited for a pilot trial of brief interventions to reduce substance use, which were delivered just prior to release from jail. After their release, participants completed the Barriers to Treatment Inventory (BTI), which included 25 numerical items and one open-ended question on additional barriers that provided qualitative data. We described frequency of quantitative responses and qualitatively coded open-ended data using seven previously identified domains of the BTI.

**Results:**

The most commonly reported barriers assessed quantitatively were items related to Absence of Problem: “I do not think I have a problem with drugs” (42.8%), Privacy Concerns: “I do not like to talk about my personal life with other people” (35.8%), and Admission Difficulty: “I will have to be on a waiting list for treatment” (28.6%). Items related to Negative Social Support (e.g., “Friends tell me not to go to treatment”) were rarely endorsed in this sample. Responses to the open-ended question also related to Absence of Problem, Privacy Concerns, and Admission Difficulty. Additional categories of barriers emerged from the qualitative data, including Ambivalence and Seeking Informal Assistance.

**Conclusions:**

In this small sample of adults with a substance use disorder recently released from jail, barriers to treatment were frequently endorsed. Future research on larger samples is needed to understand barriers to treatment specific to justice-involved populations. Clinicians may consider using open-ended questions to explore and address barriers to addiction treatment among individuals with current or recent justice involvement.

## Background

Substance use disorders (SUD) are very common among those incarcerated by the criminal justice system: more than half of people in prison (58%) and nearly two-thirds of people in jail (63%) meet criteria for a SUD [1]. Individuals transitioning from jail or prison back into the community face many challenges, including being required to meet probation or parole conditions and expectations, reestablishing housing and employment that were lost while incarcerated, and stigma [2–5]. Those with a SUD also must contend with increased risk of mortality and returning to environments that increase the likelihood of substance use [2, 4, 6–8]. Additionally, 41% of all probationers are required to attend alcohol or drug treatment [9]. Although there is some evidence that legal pressure may improve retention in treatment, namely for those in short- or long-term residential treatment [10], many individuals may not seek treatment at all and still will drop out of outpatient treatment prematurely, despite severe consequences. For example, in a sample of 926 individuals who opted to attend addiction treatment in lieu of jail or prison, 59% dropped out of treatment, of whom 25% were reincarcerated [11]. Addiction care specifically geared towards the needs of justice-involved individuals may improve treatment engagement and mitigate retention challenges, reduce risk for returning to substance use, and help to prevent individuals’ continued involvement with the justice system [2].

In the general population of U.S. residents 12 years and older, approximately 8.1% have a SUD, but only 11–14% of those with a SUD seek or get the treatment they need [12, 13]. Multiple barriers to treatment have been identified in general samples of persons with a SUD not specific to those who are justice-involved. For example, among two large, national samples of individuals with a SUD in the United States who were not seeking treatment [12, 14] and a sample of individuals with a SUD presenting to treatment, [15] barriers included not being ready to stop using alcohol or drugs, lacking resources (e.g., insurance), and not knowing where to seek treatment [12, 14, 15].

Individuals with a SUD who are justice involved face similar treatment barriers to individuals with a SUD more generally, including lack of health care and costs of treatment [5, 16] and not seeing their substance use as a problem [3, 5, 17]. These barriers were experienced across various groups of justice-involved individuals not in SUD treatment, including those who were incarcerated [7] or on probation or parole [5, 17] and those with both alcohol and other drug use disorders [3, 5]. Despite these similarities, justice-involved individuals with a SUD appear to face additional barriers, including stigma related to having a legal history [3, 7] or criminal justice agencies’ preference to provide “drug-free treatment” that exclude pharmacotherapies for SUD [18].

However, there are limitations of prior studies of barriers to addiction treatment for justice-involved individuals. For example, some of these studies focused on specific subgroups, such as those with a drug use disorder [17] or women [7], when justice-involved individuals comprise a heterogeneous population. Other studies only examined barriers to medication-assisted treatments [18, 19]. Further, studies often queried participants on a finite selection of barriers rather than using open-ended questions (e.g., [5, 7]), which may not capture the full range of barriers experienced by this group. Given the low rates of treatment receipt among justice-involved individuals with a SUD and the subsequent consequences they may experience (e.g., re-arrest or reincarceration), there is a need for a more comprehensive understanding of barriers to addiction treatment among this group to inform more effective interventions that improve treatment retention and outcomes, and decrease recidivism. Thus, exploration of a broader range of barriers to treatment among justice-involved individuals with a SUD may be needed to help treatment programs and court systems reduce these barriers and/or directly address them with their clients.

To improve our understanding of barriers to addiction treatment experienced by justice-involved individuals with a SUD more generally, the purpose of the current pilot study was to describe barriers to addiction treatment reported among a small sample of recently incarcerated individuals with a SUD, including responses provided to an open-ended question of additional barriers. Given the small sample size and broad assessment of barriers to treatment that has not been done previously with justice-involved individuals with a SUD specifically, there were no a priori hypotheses and the study was exploratory.

## Methods

### Data source

The parent study took place between April 2014 and June 2015. The current study represents a secondary analysis of follow-up data collected from a pilot randomized clinical trial of a single, 1-h brief intervention to reduce substance use with participants who were recruited from a large detention center in the Southwestern United States. The pilot trial was advertised as a study on preparing for release from jail; thus, individuals were not necessarily treatment-seeking. Individuals were recruited while they were incarcerated via flyers and presentations, and all screening, informed consent, baseline assessments, and intervention procedures took place prior to their release from jail. Participants in the trial received either a motivational intervention to reduce substance use or an educational intervention on substance use and addiction within 14 days of their pending release from jail [20]. Participants were contacted to complete two follow-up interviews after their release from jail including a 1-month interview and a 3 to 6-month interview. All participants signed a consent form. All study procedures were approved by an institutional review board at the University of New Mexico.

The parent study enrolled 40 participants, of whom 25 completed at least one follow-up interview [20]. There were an additional 3 participants consented into the parent trial who completed a follow-up interview but were ultimately excluded from the parent trial analyses for various reasons (e.g., female participants were excluded from main analyses to reduce heterogeneity). The present study included these three additional participants for a total of 28 participants who completed at least one follow-up interview and, thus, responded to questions regarding barriers to treatment. Participants who completed a follow-up interview did not differ from those who did not on demographic characteristics, pre-incarceration substance use, or lifetime treatment or legal histories. For participants who completed both follow-up interviews, quantitative data from their 1-month interview only were used; qualitative responses were used from both interviews to maximize data.

### Participants

Recruitment targeted individuals with an alcohol- or drug-related charge (e.g., driving under the influence, possession of a controlled substance), who were sentenced and had a release date, and agreed to participate in an in-person follow-up interview after their release. Of those who met these criteria, eligible individuals were further screened for: (a) having “moderate to high alcohol involvement” in the 3 months prior to their current incarceration as assessed by the Alcohol, Smoking, and Substance Involvement Screening Test (scores of 4 and above) [21], and (b) being released within 14 days. Exclusion criteria were: (a) scoring above 20 on the Mini Mental Status Exam [22], suggesting gross cognitive impairment; (b) reporting active psychotic symptoms as assessed by the Structural Clinical Interview for DSM-IV Diagnoses psychotic screening [23]; (c) lacking English proficiency; and (d) participating in the methadone maintenance program at the detention center, as they had more clinical contact throughout their incarceration than other individuals.

### Data collection

At the baseline interview prior to their release, participants completed a battery of assessments (e.g., demographic information, treatment history) and provided post-release location information, including phone numbers, mailing addresses, and email addresses for themselves, friends and family members, and anyone else with whom they might have contact. Once released, participants were contacted to complete a 1-month interview (28–49 days post-release) and a 3 to 6-month interview (84–180 days post-release), both of which were conducted in-person. If participants were unable to complete the 1-month interview within the designated timeframe, they still were eligible to complete the 3 to 6-month interview.

Participants’ blood alcohol contents were tested prior to beginning follow-up interviews to ensure they were less than 0.02 before proceeding. They then completed assessments that included substance use and treatment seeking behaviors since being released and barriers to treatment, and were compensated for their time ($25 for 1-month interview, $40 for 3 to 6-month interview).

### Measures

#### Demographic information

Demographic information was assessed at the baseline interview. Participants’ gender, age, race/ethnicity, and other demographic information were measured using the CASAA Demographics Interview form [24]. Diagnostic criteria for lifetime and current (12 months prior to baseline interview) alcohol use disorder and up to four non-alcohol SUDs were assessed using the Structural Clinical Interview for DSM-IV Diagnoses Substance Use Disorder module [23]. Participants also completed the Alcohol and Drug Use sections of the Addiction Severity Index [25], which provided information on lifetime alcohol and drug treatment episodes.

#### Substance use and treatment seeking

At the follow-up interviews, the Form-90 [26] was used to measure the percentage of days after release from jail without any use of alcohol or drugs. The parent study added an interview question to the Form-90 that queried any substance use treatment seeking behaviors (yes/no) since release from jail (“Any substance use treatment seeking behaviors?”).

#### Barriers to treatment

Barriers were assessed at the follow-up interviews with the Barriers to Treatment Inventory (BTI) [15]. The BTI is a seven factor, 25-item self-report measure and individuals rated agreement with potential barriers (for list of items, see Table 1) on a 1–5 Likert-type scale (1 = Strongly Disagree, 5 = Strongly Agree). Rapp and colleagues [15] administered the full 59-item BTI to 312 individuals with a SUD presenting to an addiction treatment intake center and conducted the initial factor analysis. Of the original 59 items, a seven-factor model was found to have adequate fit and was comprised of 25 items [15]. The seven factors included: Absence of Problem (e.g., “I do not think I have a problem with drugs”, “I do not think treatment will make my life better”), Negative Social Support (“I will lose my friends if I go to treatment”, “My family will be embarrassed or ashamed if I go to treatment”), Fear of Treatment (“I have had a bad experience with treatment”, “I am too embarrassed or ashamed to go to treatment”), Privacy Concerns (“I do not like to talk in groups”, “I do not like to talk about my personal life with other people”), Time Conflict (“It will be hard for me to find a treatment program that fits my schedule”), Poor Treatment Availability (“I am moving too far away to get treatment”, “I have difficulty getting to and from treatment”), and Admission Difficulty (“I have to go through too many steps to get into treatment”) [15]. Cronbach’s alphas for each factor domain ranged from 0.65 to 0.86 [15]. For the current study, responses to each item on the BTI were grouped into three types: Disagree (ratings of 1 or 2), Uncertain (rating of 3), and Agree (ratings of 4 or 5).

**Table 1:** Sample characteristics among 28 adults with a SUD recently released from jail

	Mean (SD)	*n* (%)
Age (years)	35.1 (10.1)	
Male		27 (96.4%)
*Race/ethnicity*		
Hispanic		7 (25.0%)
Biracial/multiracial		7 (25.0%)
American Indian/Alaskan native		6 (21.4%)
Non-Hispanic White		5 (17.9%)
Other		3 (10.7%)
Days Incarcerated*	168.5 (106.9)	
Any current substance use disorder**		23 (85.2%)
Current alcohol use disorder**		19 (70.4%)
Current drug use disorder**		14 (51.9%)
Lifetime alcohol use disorder		28 (100.0%)
Lifetime drug use disorder		26 (92.9%)
Lifetime alcohol treatment episodes	3.9 (5.2)	
Lifetime drug treatment episodes	2.1 (3.1)	
Post-release percent days abstinent	67.9 (38.4)	
Post-release treatment seeking (formal or informal)		10 (35.7%)

*Data for *n *= 2 participants were removed because they were incarcerated for more than 2 SD from the mean (range = 31–874 days)

**Data were available for *n *= 27 participants only. Of the 27 participants with complete diagnostic data, the *n *= 4 participants who did not meet criteria for any current substance use disorder all had been incarcerated in jail for more than 12 months

One open-ended question was added to the end of the BTI that asked, “Please describe any additional barriers to treatment in your own words.” Participants provided written responses to this question.

### Data analyses

Sample characteristics were described overall. The mean and standard deviation for each BTI item, as well as the *n* and percent that endorsed each response type (Disagree, Uncertain, Agree) were calculated. Each individual BTI item was described (rather than aggregated in previously identified BTI domains [15]) in order to describe the full range of barriers reported in this sample for this hypothesis-generating study. We then coded qualitative responses to the open-ended question of additional barriers using template analysis [27] (i.e., content analysis or thematic coding). In this approach, data are coded both inductively and deductively, thus allowing for coding of both a priori domains and emergent domains. The initial coding template was based on the seven factors (domains) of the BTI reported by Rapp et al. [15]. Two coders (M.D.O., J.A.C.) independently coded open-ended responses; discrepancies then were discussed by all authors until consensus was reached. Consistent with the recommendations by Sandelowski [28], we opted not to report frequencies of qualitative results, as the use of numbers may have detracted from making well-rounded interpretations of the data, particularly in the context of data generated from a single, open-ended survey question as opposed to an in-depth interview.

## Results

Among the 28 participants who completed at least one follow-up interview, 11 completed only the 1-month interview, 6 completed only the 3 to 6-month interview, and 11 completed both. Responses to the open-ended question of additional barriers were examined for all completed interviews (*n *= 39), of which four were left blank (10.3%). Just over half of participants included in the present study were randomized to the motivational intervention condition (*n *= 15, 53.6%). Sample characteristics are provided in Table 1.

The means and standard deviations, as well as the number and percentage of participants endorsing items in each of the seven BTI domains are provided in Table 2. The most commonly endorsed barriers to addiction treatment were related to the domain Absence of Problem: “I do not think I have a problem with drugs” (42.8%) and “My drug use is not causing any problems” (32.2%), and Privacy Concerns: “I do not like to talk about my personal life with other people” (35.7%) and “I do not like to talk in groups” (32.2%). No participants endorsed four of the five barriers from the Negative Social Support domain: “I will lose my friends if I go to treatment”, “People will think badly of me if I go to treatment,” “Someone in my family does not want me to go to treatment,” or “My family will be embarrassed or ashamed if I go to treatment.”

**Table 2:** Descriptive information from the Barriers to Treatment Inventory

	Mean	SD	Disagree *n* (%)	Uncertain *n* (%)	Agree *n* (%)
*Absence of Problem*	2.6	1.1			
I do not think I have a problem with drugs	3	1.5	12 (42.8)	4 (14.3)	12 (42.8)
No one has told me I have a problem with drugs	2.5	1.5	17 (60.7)	3 (10.7)	8 (28.6)
My drug use is not causing any problems	2.7	1.6	15 (53.5)	4 (14.3)	9 (32.2)
I do not think treatment will make my life better	2.3	1.2	18 (64.3)	6 (21.4)	4 (14.3)
I can handle my drug use on my own	2.9	1.4	12 (42.8)	8 (28.6)	8 (28.6)
I do not think I need treatment	2.5	1.3	17 (60.7)	4 (14.3)	7 (25.0)
*Negative Social Support*	1.8	0.5			
I will lose my friends if I go to treatment	1.8	0.7	23 (82.1)	5 (17.9)	0
Friends tell me not to go to treatment	2.0	1.0	23 (82.1)	2 (7.1)	3 (10.7)
People will think badly of me if I go to treatment	1.8	0.6	25 (89.3)	3 (10.7)	0
Someone in my family does not want me to go to treatment	1.6	0.6	26 (92.9)	2 (7.1)	0
My family will be embarrassed or ashamed if I go to treatment	1.5	0.6	26 (92.9)	2 (7.1)	0
*Fear of Treatment*	1.9	0.8			
I have had a bad experience with treatment	2.1	1.2	21 (75.0)	2 (7.1)	5 (17.8)
I am afraid what might happen in treatment	1.9	0.9	23 (82.1)	3 (10.7)	2 (7.1)
I am afraid of the people I might see in treatment	1.8	0.9	23 (82.1)	3 (10.7)	2 (7.1)
I am too embarrassed or ashamed to go to treatment	1.8	0.9	23 (82.1)	3 (10.7)	2 (7.1)
*Privacy Concerns*	2.7	1.2			
I do not like to talk in groups	2.6	1.4	18 (64.3)	1 (3.6)	9 (32.2)
I hate being asked personal questions	2.6	1.3	17 (60.7)	3 (10.7)	8 (28.6)
I do not like to talk about my personal life with other people	2.8	1.3	16 (57.2)	2 (7.1)	10 (35.7)
*Time Conflict*	2.2	0.9			
I have things to do at home that make it hard for me to get to treatment	2.1	1.0	21 (75.0)	3 (10.7)	4 (14.3)
It will be hard for me to find a treatment program that fits my schedule	2.3	1.0	17 (60.7)	8 (28.6)	3 (10.7)
*Poor Treatment Availability*	2.0	0.9			
I am moving too far away to get treatment	2.0	1.0	23 (82.1)	3 (10.7)	2 (7.1)
I do not know where to go for treatment	1.9	0.8	22 (78.6)	5 (17.9)	1 (3.6)
I have difficulty getting to and from treatment	2.1	1.2	20 (71.4)	2 (7.1)	6 (21.5)
*Admission Difficulty*	2.6	1.1			
I will have to be on a waiting list for treatment	2.7	1.2	12 (42.8)	8 (28.6)	8 (28.6)
I have to go through too many steps to get into treatment	2.5	1.1	16 (57.1)	6 (21.4)	6 (21.5)

*1* Strongly disagree, *3* uncertain, *5* strongly agree. “Disagree” includes responses to both “Disagree” and “Strongly Disagree”. “Agree” includes responses to both “Agree” and “Strongly Agree”

Results of the qualitative data analysis are presented in Table 3. Coding identified responses consistent with a priori domains based on Rapp et al.’s factor analysis [15] as well as emergent domains. Within a priori domains, barriers related to Absence of Problem (“Don’t need it”), Privacy Concerns (“I’m not completely comfortable with speaking in a group setting”), and Time Conflict (“Just pending court dates”) were endorsed. Three emergent domains were identified in the data, including Ambivalence (e.g., “The fact that I enjoy the drug”), Seeking Informal Assistance (e.g., “I have yet to go to treatment cause [sic] I want to try and see if the support im [sic] getting from my family will be enough…”), and Other, which included open-ended responses that were difficult to interpret or code (e.g., “AA meetings” and “The fact that alcohol is advertised all around”).

**Table 3:** Examples of additional barriers identified in responses to open-ended question, organized by a priori and emergent domains

a Priori domains	Examples of responses from open-ended question
Absence of Problem	“Don’t need it” “I just don’t want to go to treatment. I’m fine on my own”
Negative Social Support	None listed
Fear of Treatment	“Don’t want to, it hasn’t helped in the past” “I just don’t like the sound of it”
Privacy Concerns	“I’m not completley comfortable with speaking in a group setting” “Myself—hard to get myself open like that (hard to trust)”
Time Conflict	“Just pending court dates” “Not wanting to take the time to go”
Poor Treatment Availability	“Transportation and time avail. Ex: You have a 8 am drug treatment class in Bernalillo Cty-while I now live in Santa Fe city [sic]” “Transportation is an issue”
Admission Difficulty	“Just my misdemeanor situation prevents inpatients treatments to accept me. That’s about it as far as I can see”
*Emergent domains*	
Ambivalence	“I don’t have the motivation to go. I’m lazy” “The fact that I enjoy the drug”
Seeking Informal Assistance	“I have yet to go to treatment cause [sic] I want to try and see if the support im [sic] getting from my family will be enough”
Other	“AA meetings” “The fact that alcohol is advertised all around” “I have heard it all”
No barriers	“Currently attending treatment. I am ready to change my life for the better” “Currently I have no barriers”

Each response was coded using the barrier factor domains reported by Rapp et al. [15]. Responses that did not fit into an existing category were coded as emergent domains. Responses to open-ended questions came from all completed interviews (*n *= 39), of which four were left blank. One response included multiple barriers, so this was coded into two domains

### Post-hoc analyses

To explore potential influences of reported barriers to treatment, independent samples *t* tests were conducted to examine between-group differences in endorsements of BTI items across study condition (from the parent trial, motivational intervention: *n *= 15, educational intervention: *n *= 13) and between those who did (*n *= 10) and did not (*n *= 18) report engaging in any treatment seeking behaviors after release from jail. Because we considered these between-group comparisons exploratory and not hypothesis-driven, there were no a priori power analyses conducted, and results are to be used for hypothesis generation.

Exploratory comparisons across study conditions suggested that, relative to those who received the educational intervention, participants randomized to receive the motivational intervention were more likely to agree with barriers related to Absence of Problem: “I do not have a problem with drugs” (*M *= 3.5, *SD *= 1.3 vs. *M *= 2.4, *SD *= 1.5; *t *= 2.169, *p *< .05) and “I can handle my drug use on my own” (*M *= 3.4, *SD *= 1.4 vs. *M *= 2.2, *SD *= 1.1; *t *= 2.491, *p *< .05; see Appendix A). There were no other between group differences by study condition identified in exploratory analyses.

Participants who reported seeking any post-release treatment appeared less likely than those who did not seek treatment to endorse one barrier from Absence of Problem: “I do not think treatment will make my life better” (*M *= 1.6, *SD *= 0.7 vs. *M *= 2.7, *SD *= 1.2; *t *= 2.501, *p *< 0.05), and two barriers related to Privacy Concerns: “I do not like to talk in groups” (*M *= 1.9, *SD *= 0.9 vs. *M *= 3.0, *SD *= 1.5; *t *= 2.454, *p *< 0.05) and “I hate being asked personal questions” (*M *= 1.9, *SD *= 0.7 vs. *M *= 3.1, *SD *= 1.3; *t *= 2.993, *p *< 0.05; see Appendix B). No other differences in reported barriers to treatment among those who sought versus did not seek treatment after release from jail were identified in post hoc analyses.

## Discussion

This exploratory study used a survey with closed- and open-ended responses to describe barriers to addiction treatment in a small sample of recently-incarcerated adults with a SUD, a population among whom addiction treatment may be of paramount importance, is underutilized, and rarely tailored to their needs. Barriers to addiction treatment were frequently endorsed, but there were no barriers universally endorsed by this sample. Findings can be considered hypothesis-generating regarding barriers to treatment that might be specific to this population. These findings may be useful in informing future research and clinical efforts for individuals with a SUD with recent or current justice involvement.

Previous studies among justice-involved populations have identified lack of healthcare and related costs, limited availability of qualified healthcare providers, long wait times, stigma, and criminal justice agencies’ procedures and preferences (e.g., exclusion of medication-assisted treatments due to preference to be “drug-free”) as barriers to treatment [4, 5, 7, 18, 19, 29]. There were similarities in findings between studies on barriers to treatment with other justice-involved samples and the current study. Among justice-involved individuals choosing to attend treatment in lieu of incarceration, [17] 23.9% expressed no desire for treatment, which is similar to 25.0% of the current sample reporting “I do not think I need treatment”. Rose et al. (2014) found that women incarcerated in jail also reported long wait lists as a barrier to treatment, although this was more common among this sample of women than the current sample (58.9% vs. 28.6%) [7]. Unfortunately, it often is difficult to make comparisons across studies due to variability in assessing barriers and differing samples. The present study is small, but potentially broader in inclusion criteria than previous studies, and adds to these previous studies by expanding the assessment of barriers. Indeed, we found that barriers not previously identified may be important to consider for this population, including perceiving Absence of Problem and Privacy Concerns. Knowledge of these potential barriers in combination with those previously identified in justice-involved individuals, such as Ambivalence, may provide counselors and criminal justice staff with more nuanced information to assist their clients in overcoming obstacles to seeking or staying in treatment.

A previous study also used the BTI, but with a sample of general treatment-seeking individuals with a SUD, [15] which presented the opportunity to compare rates of barriers to the current sample of justice-involved individuals with a SUD. Among this sample of treatment-seeking individuals, barriers to treatment included the Fear of Treatment, Time Conflict, and Poor Treatment Availability, which also were endorsed by similar proportions of the current study sample. It is noteworthy, however, that participants in the present study sample endorsed Absence of Problem at a markedly higher rate than what was reported by Rapp et al., such as, “I do not think I have a problem with drugs” (42.8% vs. 12.5%) and “My drug use is not causing any problems” (32.2% vs. 8.0%). These discrepancies may be because the sample from Rapp et al. already had initiated the process of seeking addiction treatment, which often includes an acknowledgement of engaging in problematic substance use, compared to the current sample, who were not necessarily treatment-seeking. Alternatively, the present study selected for people reporting moderate to high alcohol use who may not have had much drug involvement. Because we did not alter the original scale to include both drugs and alcohol in these items, we may have inadvertently overestimated the degree to which our participants felt they had an “Absence of Problem.” However, half of the sample met criteria for a current drug use disorder, suggesting that many individuals in this sample still were experiencing problems related to their drug use.

Though our sample was small, an interesting finding was the relatively few endorsements of barriers in the Negative Social Support domain. Specifically, participants in the present study did not endorse four of the five items, and only a small number endorsed “Friends tell me not to go to treatment” (*n *= 3, 10.7%). In contrast, these barriers were more frequently endorsed by the treatment-seeking sample of individuals with a SUD reported by Rapp et al., although still in low proportions (proportions of agreement ranged from 7.4 to 16.0%) [15]. This finding suggests the possibility that justice-involved individuals with a SUD experience barriers related to Negative Social Support less frequently. While future research is needed to further explore this finding, these results suggest a possible intervention pathway—via the involvement of friends and family to encourage treatment engagement. Additionally, these findings may indicate that resources should be diverted to addressing other barriers, where they may have more impact.

In exploratory, post hoc analyses comparing barriers between participants randomly assigned to treatment conditions, barriers generally were endorsed by similar proportions. Groups did, however, differ on two items from the Absence of Problem domain (“I do not have a problem with drugs”, “I can handle my drug use on my own”), with participants assigned to the motivational intervention reporting these barriers more frequently than those assigned to the education intervention. These findings are hypothesis-generating in suggesting that motivational interventions may not be helpful for increasing problem recognition among justice-involved individuals with a SUD, as they are designed to do, or may be unhelpful in this domain. Other studies with jail populations found null results of motivational interventions on other outcomes such as treatment engagement [30]. Of note, our results should be interpreted with caution, given the small sample, post hoc nature of these analyses, multiple comparisons, and that the parent study did not include a no-treatment control condition.

Similarly, in post hoc analyses comparing barriers reported between groups based on post-release treatment seeking, groups largely were similar. However, those who reported seeking treatment reported less apprehension about talking in groups or being asked personal or intrusive questions as a barrier. These findings highlight privacy as a potential concern among those who did not seek treatment, which may have clinical implications. This non-treatment-seeking group may prefer individual treatment or find pharmacotherapy less invasive and more beneficial than psychotherapy. However, research is needed in larger samples of justice-involved adults with a SUD who have and have not sought treatment to further explore this issue, and examine if pharmacotherapy could be a viable option for those with Privacy Concerns.

Findings from the present study suggest the possibility that barriers beyond those commonly identified may exist in this population. Two participants specifically referenced issues related to their justice involvement in the reporting of additional barriers that were coded under a priori domains (“Just pending court dates” coded as Time Conflict; and “Just my misdemeanor situation prevents inpatients treatments to accept me” coded as Admission Difficulty). Though justice-involvement was not, on its own, coded as an emergent barrier domain, it is possible that this population experiences specific enhanced barriers to treatment that were not cued by the generally phrased open-ended question we used regarding potential additional barriers to treatment. Additionally, it would be useful to better understand potential barriers identified with more in-depth qualitative work. For example, it would be informative to know if “pending court dates” refer to multiple demands on time or waiting to start treatment to know exact sentencing requirements, as well as to know how a “misdemeanor situation” may prevent someone from entering inpatient treatment (e.g., if someone is precluded from treatment while they wait for court dates). We had limited ability to assess these issues due to both small sample size and administration of one open-ended question without the opportunity to probe. Nonetheless, our study is hypothesis-generating regarding the need for broadening measures of barriers to treatment for persons involved with the criminal justice system, which can then inform future interventions for this population.

The present study is limited in several additional ways. First, this study had a small sample and thus the results presented here should be considered descriptive and hypothesis-generating. Given the small sample, we likely were underpowered to detect between-group differences across treatment condition assignment and report of post-release treatment receipt. The multiple exploratory comparisons we conducted between groups increase the risk of making a Type II error. Moreover, findings may not be representative of justice-involved individuals with a SUD more generally (e.g., those who are arrested without ever being incarcerated) because the sample only included individuals who were sentenced, had a substance use-related charge, were being released from a large Southwest detention center, and agreed to participate in a clinical trial, and recruitment targeted those with an alcohol use disorder (e.g., some may not have identified with needing “drug treatment”); this study also excluded those in the methadone maintenance program. The study recruited for individuals with an alcohol use disorder, but many questions of the BTI use language referring to drug use specifically (e.g., “I do not think I have a problem with drugs”), which may have influenced responses to these BTI items and resulted in overestimates of these barriers. Findings also may not be generalizable to women, and there may be additional barriers experienced by women not reflected in these results (e.g., competing basic needs related to childcare, added stigma [7]). Findings also may be biased by loss to follow-up. Though we compared participants across follow-up status and identified no differences, participants who did not complete either follow-up interview may experience more or different barriers to treatment (e.g., lacking reliable access to a phone, which may make it difficult to seek out treatment services). Further, many participants already had made changes to their alcohol and/or drug use by the time they completed the follow-up interview, which may have influenced their perceived need for treatment. Because this study was a secondary analysis of a pilot parent study that was not designed to investigate issues related to barriers to treatment, we may have missed some barriers to treatment (e.g., barriers to addiction treatment related to cost and lack of healthcare coverage did not emerge), which are commonly reported in the literature [5, 16]. We also did not assess some key issues that may influence barriers to treatment, such as conditions of release or probation or parole requirements (e.g., having more probation appointments interfering with ability to attend treatment) or provide external incentives to engage in treatment (e.g., SUD treatment is a condition of probation and compliance is associated with remaining out of jail or prison). Finally, participants were not given prompts or further opportunity to expand on potential barriers to treatment, as the BTI is a self-report measure rather than an interview.

## Conclusions

Though this study is small, it contributes to the burgeoning literature on barriers to addiction treatment in a sample of individuals with a SUD with recent or current justice involvement. Results suggest that although barriers reported were variable across the sample and none were endorsed uniformly, recently incarcerated individuals with a SUD experience many barriers to treatment. While some barriers may be similar to those experienced by non-justice-specific populations with a SUD, findings from the present study indicate the possibility of unique barriers within this population of recently incarcerated adults. Specifically, it is possible that this population experiences greater barriers related to not seeing their substance use as a problem and fewer barriers related to Negative Social Support. There also may be barriers to treatment that are justice-specific, and these barriers may be missed by commonly-used instruments, such as the BTI. Results indicate a strong need for more research in larger samples to more comprehensively assess barriers in this population. Further, referring justice-involved individuals to the same addiction treatment designed for the general population (e.g., predominately group therapy) may result in barriers for them (e.g., not wanting to talk in groups) and be less effective. Clinicians working with justice-involved individuals with a SUD may want to use open-ended questions to explore potential barriers to addiction treatment, so that they may anticipate and plan for them. Better understanding barriers to treatment experienced by justice-involved individuals with a SUD is a promising area for research as it will ultimately help to inform new interventions aimed at increasing receipt of addiction treatment for this population.

## Appendix A: Post-hoc results of between-group differences by study condition

**Table:**

	Motivational		Educational		*t*-value
	Mean	SD	Mean	SD	
*Absence of Problem*	3.0	1.0	2.3	1.1	1.758
I do not think I have a problem with drugs	3.5	1.3	2.4	1.5	2.169*
No one has told me I have a problem with drugs	2.7	1.4	2.3	1.5	0.645
My drug use is not causing any problems	3.0	1.6	2.3	1.5	1.176
I do not think treatment will make my life better	2.5	1.1	2.0	1.2	1.201
I can handle my drug use on my own	3.4	1.4	2.2	1.1	2.491*
I do not think I need treatment	2.7	1.2	2.3	1.4	0.866
*Negative Social Support*	1.7	0.5	1.9	0.5	1.128
I will lose my friends if I go to treatment	1.7	0.6	2.0	0.8	1.228
Friends tell me not to go to treatment	1.7	0.8	2.2	1.2	1.282
People will think badly of me if I go to treatment	1.8	0.7	1.8	0.6	0.195
Someone in my family does not want me to go to treatment	1.5	0.5	1.8	0.7	1.663
My family will be embarrassed or ashamed if I go to treatment	1.6	0.6	1.5	0.7	0.566
*Fear of Treatment*	1.8	0.6	2.0	0.9	0.760
I have had a bad experience with treatment	2.0	1.0	2.3	1.5	0.648
I am afraid what might happen in treatment	1.7	0.6	2.2	1.1	1.503
I am afraid of the people I might see in treatment	1.8	0.8	1.8	1.1	0.087
I am too embarrassed or ashamed to go to treatment	1.7	0.8	1.8	1.1	0.319
*Privacy Concerns*	2.3	0.9	3.1	1.4	1.681
I do not like to talk in groups	2.3	1.0	3.0	1.7	1.364
I hate being asked personal questions	2.3	0.9	3.0	1.5	1.380
I do not like to talk about my personal life with other people	2.3	1.0	3.2	1.4	1.876
*Time Conflict*	2.1	0.9	2.2	0.9	0.173
I have things to do at home that make it hard for me to get to treatment	2.0	1.1	2.2	1.0	0.393
It will be hard for me to find a treatment program that fits my schedule	2.3	0.9	2.2	1.1	0.096
*Poor Treatment Availability*	1.9	1.0	2.1	0.7	0.500
I am moving too far away to get treatment	1.9	1.1	2.1	0.9	0.570
I do not know where to go for treatment	1.9	1.0	1.8	0.7	0.503
I have difficulty getting to and from treatment	1.9	1.2	2.4	1.3	0.960
*Admission Difficulty*	2.6	1.0	2.5	1.2	0.222
I will have to be on a waiting list for treatment	2.9	1.3	2.5	1.2	0.690
I have to go through too many steps to get into treatment	2.4	1.1	2.5	1.3	0.316

*n *= 15 received Motivational Intervention (treatment condition); *n *= 13 received Educational Intervention (control condition)

*1* Strongly disagree, *3* Uncertain, *5* Strongly agree

**p *< 0.05

## Appendix B. Post-hoc results of between-group differences by post-release treatment seeking

**Table:**

	Sought treatment		Did not seek treatment		*t*-value
	Mean	SD	Mean	SD	
*Absence of Problem*	2.3	0.9	2.9	1.2	1.403
I do not think I have a problem with drugs	2.9	1.6	3.1	1.5	0.260
No one has told me I have a problem with drugs	2.1	1.3	2.7	1.5	1.090
My drug use is not causing any problems	2.7	1.6	2.7	1.6	0.053
I do not think treatment will make my life better	1.6	0.7	2.7	1.2	2.501*
I can handle my drug use on my own	2.3	1.3	3.2	1.3	1.678
I do not think I need treatment	1.9	1.0	2.9	1.3	2.056
*Negative Social Support*	1.7	0.6	1.8	0.5	0.571
I will lose my friends if I go to treatment	1.7	0.7	1.9	0.8	0.656
Friends tell me not to go to treatment	1.5	0.5	2.2	1.2	1.845
People will think badly of me if I go to treatment	1.9	0.7	1.8	0.5	0.499
Someone in my family does not want me to go to treatment	1.6	0.7	1.7	0.6	0.267
My family will be embarrassed or ashamed if I go to treatment	1.7	0.8	1.4	0.5	0.891
*Fear of Treatment*	2.0	0.9	1.9	0.7	0.247
I have had a bad experience with treatment	2.5	1.4	1.9	1.1	1.144
I am afraid what might happen in treatment	1.9	1.0	1.9	0.8	0.032
I am afraid of the people I might see in treatment	1.8	1.0	1.8	0.9	0.060
I am too embarrassed or ashamed to go to treatment	1.6	0.7	1.9	1.0	0.793
*Privacy Concerns*	2.1	0.8	3.0	1.3	2.251*
I do not like to talk in groups	1.9	0.9	3.0	1.5	2.454*
I hate being asked personal questions	1.9	0.7	3.1	1.3	2.993**
I do not like to talk about my personal life with other people	2.5	1.3	2.9	1.3	0.756
*Time Conflict*	1.9	0.9	2.3	0.9	1.173
I have things to do at home that make it hard for me to get to treatment	1.9	1.0	2.2	1.0	0.659
It will be hard for me to find a treatment program that fits my schedule	1.9	0.9	2.4	1.0	1.457
*Poor Treatment Availability*	1.7	0.7	2.1	0.9	1.336
I am moving too far away to get treatment	1.7	0.7	2.1	1.1	1.088
I do not know where to go for treatment	1.6	0.8	2.0	0.8	1.206
I have difficulty getting to and from treatment	1.8	1.0	2.3	1.3	1.096
*Admission Difficulty*	2.5	1.2	2.6	1.1	0.313
I will have to be on a waiting list for treatment	2.7	1.4	2.7	1.2	0.044
I have to go through too many steps to get into treatment	2.3	1.1	2.6	1.2	0.562

*n *= 10 reported seeking any type of treatment after release from jail; *n *= 18 reported that they did not seek any type of treatment after release

*1* Strongly disagree, *3* Uncertain, *5* Strongly agree

**p *< 0.05; ***p *< 0.01
